# Will histogram analysis replace mean attenuation threshold in the diagnosis of adrenal lesions?

**DOI:** 10.1590/0100-3984.2023.56.2e1

**Published:** 2023

**Authors:** Refky Nicola

**Affiliations:** 1 Associate Professor of Radiology, Division of Abdominal Radiology, SUNY Upstate Medical University, Syracuse, NY, USA. Email: rnicola04@gmail.com

The article “Histogram analysis in the differentiation between adrenal adenomas and pheochromocytomas: the value of a single measurement”, authored by Teixeira et al.^(1)^ and published in this issue of Radiologia Brasileira, compares the diagnostic accuracy of histogram analysis with the standard technique of using the mean attenuation threshold for a diagnostic assessment of adrenal adenomas and pheochromocytomas. As early as 1992, it was reported by Lee et al. the use of Hounsfield attenuation value for lipid rich adrenal adenoma measures less than 10 HU^(2)^. This has become the standard practice for the diagnosis of adrenal adenomas. However, this methodology is not without its own set of shortcomings.

Even though the prevalence of pheochromocytomas account for only 3–7% of all adrenal incidentalomas as per reported in the article, a significant portion of pheochromocytomas is either mild symptomatic or completely asymptomatic. In addition, a large portion of adrenal adenoma may demonstrate a mean attenuation > 10 HU. These types of lesions are considered a lipid poor adenoma. While the 15-minute washout technique may resolve the predicament, this technique becomes inaccurate for differentiating lipid poor adrenal adenomas from pheochromocytomas since both can exhibit significant washout. These results still do not resolve this significant quandary.

Teixeira et al. utilized a method of histogram analysis of both 52 adrenal adenomas and 29 pheochromocytomas which measure less than 4.0 cm in size. This technique which was described by Bae et al.^(3)^ to differentiate between adrenal adenoma and metastasis involves the placement of a region of interest (ROI) on the unenhanced CT image of the adrenal lesion and number of pixels which measure less than 10 HU are counted. Additional studies have reported that by using this method the sensitivity and specificity can be improved if a threshold of more than 5–10% negative pixels is used for the diagnosis of an adenoma^(4,5)^. In their study, they extracted 10th percentile (P10) from the conventional histogram analysis as well as calculated by using the formula, *P10* = *mean attenuation* – (1.282 **×** *standard deviation*) and compared to the mean attenuation for each lesion. The study was conducted by two radiologist was similar years of experience. Their results show that mean attenuation threshold had a sensitivity, specificity, and accuracy lower than observed P10 and calcP10 and demonstrated statistical significance.

Even though Remer et al. have reported lower sensitivity in the evaluation of adrenal lesions, it was probably due to the inclusion of adrenal metastasis in their cohort^(5)^.

This article confronts the question of whether mean attenuation threshold is still a value tool in the diagnosis of adrenal lesions. While mean attenuation threshold is still useful for lipid rich adrenal adenomas, adrenal lesions which are potentially diagnosed as lipid poor adenomas, may warrant further investigation with histogram analysis. Although MRI may present as an alternative, patients who are incapable of undergoing MRI for reasons such as contrast reaction, pacemaker device, and length of time in the MRI scanner, a histogram analysis of the adrenal lesions may present as a viable and safe alternative.
